# Human-Specific HERV-K Insertion Causes Genomic Variations in the Human Genome

**DOI:** 10.1371/journal.pone.0060605

**Published:** 2013-04-12

**Authors:** Wonseok Shin, Jungnam Lee, Seung-Yeol Son, Kung Ahn, Heui-Soo Kim, Kyudong Han

**Affiliations:** 1 Department of Nanobiomedical Science and WCU Research Center, Dankook University, Cheonan, Republic of Korea; 2 Department of Microbiology, College of Advanced Science, Dankook University, Cheonan, Republic of Korea; 3 Department of Biological Sciences, College of Natural Sciences, Pusan National University, Busan, Republic of Korea; University of Poitiers, France

## Abstract

Human endogenous retroviruses (HERV) sequences account for about 8% of the human genome. Through comparative genomics and literature mining, we identified a total of 29 human-specific HERV-K insertions. We characterized them focusing on their structure and flanking sequence. The results showed that four of the human-specific HERV-K insertions deleted human genomic sequences via non-classical insertion mechanisms. Interestingly, two of the human-specific HERV-K insertion loci contained two HERV-K internals and three LTR elements, a pattern which could be explained by LTR-LTR ectopic recombination or template switching. In addition, we conducted a polymorphic test and observed that twelve out of the 29 elements are polymorphic in the human population. In conclusion, human-specific HERV-K elements have inserted into human genome since the divergence of human and chimpanzee, causing human genomic changes. Thus, we believe that human-specific HERV-K activity has contributed to the genomic divergence between humans and chimpanzees, as well as within the human population.

## Introduction

Repetitive mobile elements are responsible for half of the human genome. Among them, human endogenous retroviruses (HERVs) and related sequences account for ∼8% of the human genome [1]. It is thought that HERVs are derived from exogenous retrovirus infections early in the evolution of primates because they have a similar structure to the provirus of an infectious virus [2]. A full-length HERV element is approximately 9.5 kb in length and consists of an internal region of four essential viral genes (*gag*, *pro*, *pol*, and *env*) and two long terminal repeats (LTRs); *gag* stands for group-specific antigen which is the retroviral capsid protein, *pro* encodes for a protease, and *pol* contains a reverse transcriptase domain [3], [4]. HERVs are distinguished from other LTR retrotransposons by the presence of the envelope (*env*) gene, which codes for viral membrane proteins [5]. The LTRs contain many regulatory elements such as promoters, enhancers, and polyadenylation signals required for retroviral gene expression [6], [7].

Since the initial infection of HERV into its host genome, the elements have lost their ability to synthesize mature retroviral particles by accumulating mutations preventing them from infecting other cells [8]. Nonetheless, they have successfully propagated within genomes via retrotransposition and vertical inheritance, reaching ∼203,000 copies in the human genome [1]. HERVs fall into three different classes (I-III) based on sequence similarity to different genera of infectious retroviruses, and each class comprises many families with independent origins [1], [3]. There are 31 HERV families in the human genome and they are named according to the specificity of the tRNA primer-binding site [3], [9]. It was reported that most HERV families underwent radiations in their host genomes after the divergence of Old and New World monkeys [8]. Among the three HERV classes, class II HERVs exist in the lowest frequency in the human genome, but they include the HERV-K family, which is the youngest family and is known to have actively mobilized since the divergence of humans and chimpanzees [1], [10]. The HERV-K subfamily could be integrated and endogenized into the human genome by germ-line infection, which was supported by the evidence of purifying selection on the *env* gene of HERV-K elements [11].

It has been suggested that the HERV-K family is the most biologically active family because it retains the ability to encode functional retroviral proteins and produce retrovirus-like particles [12], [13], [14]. Due to this, the HERV-K family has been the subject of many studies but to date no functional provirus capable of producing infectious particles has been detected [10]. Although the HERV-K family emerged in the catarrhine lineage prior to the divergence of hominoids and Old World monkeys, some of its members inserted into the human genome after the divergence of humans and chimpanzees [8]. Thus, the HERV-K family may have contributed to the genomic differences between humans and chimpanzees through species-specific insertion and subsequent related genomic rearrangements. In this study, we identified 29 human-specific HERV-K elements in the human genome and examined the human genomic changes caused by these insertions. Our analyses focused on the mechanisms through which the HERV-K insertions caused the observed changes. In addition, we conducted a polymorphism test of the HERV-K insertions in human populations, the result of which indicates that HERV-K elements may also be contributing to genomic variations within the human species.

## Results and Discussion

### Identification of Human-specific HERV-K Insertions

To identify human-specific HERV-K elements, we first extracted 2,618 HERV-K elements from the human genome. However, some of these elements contained other internal non-HERV repeat element insertions or internal sequence deletions. In these cases, each HERV-K fragment was counted as a separate element by the tool we used to extract them, rather than counting the un-fragmented element only once. Thus, we manually inspected the HERV-K candidate loci and reassembled all fragmentary elements, resulting in a revised total of 1,390 loci (Table 1). To detect human-specific insertion loci in these 1,390 HERV-K elements, we examined the orthologous loci of each human-derived HERV-K element in the chimpanzee, orangutan, and rhesus macaque genomes. In this way, we identified 26 human-specific HERV-K loci in the human genome. Four previous studies have attempted to identify human-specific HERV-K loci [15], [16], [17], [18]. A comparison of our results showed that our strategy recovered five human-specific HERV-K loci that these previous studies missed. However, three of the human-specific HERV-K loci previously reported in the literature (HERV-K103, 113, and 134) were missing from our dataset. We examined these three loci in detail. Two were solitary LTRs in the human reference genome sequence and since we did not include solitary LTRs in our dataset of human-specific HERV-K loci, it is unsurprising these two loci were missed by our strategy. Close examination of the third missing locus revealed this locus to be polymorphic in human populations. In other words, we were unable to detect the locus because the HERV-K element is absent in the human reference genome sequence. Given this, we assert that our strategy to identify human-specific HERV-K elements in the human reference genome is robust. Thus, as shown in the Figure S1, at least 29 human-specific HERV-K elements are existed in the human genome.

**Table 1:** Summary of human-specific HERV-K insertions.

Classification	No. of loci
*Computationally predicted HERV-K loci*	1390
* Number of human-specific HERV-K insertion events*	29
Full-length human-specific HERV-K insertion	17
Truncated human-specific HERV-K insertion	8
Non-classical insertion of HERV-K	4

We characterized the human-specific HERV-K elements focusing on their size. A full-length HERV-K element consists of ∼7.5 kb of internal region and two LTRs, each of which is ∼1 kb. However, most of the HERV-K elements in the human genome contain internal deletions of variable sizes. In this study, we considered the element whose internal region is >7 kb to be a full-length element. The size of HERV-K internal regions ranged from 97 to 7546 bp, and 17 out of the 29 human-specific HERV-K elements were full-length elements according to our criterion. HERV-K elements have been grouped into two types, type I and type II, according to the presence/absence of a 292 bp sequence at the *pol-env* boundary of the elements [2]. Only type II elements contain the 292 bp sequence. We further examined the full-length human-specific HERV-K elements. As shown in Table 2, eight and nine elements are identified as type I and type II, respectively, including three previously studied insertions [16], [18].

**Table 2:** The structural characterization of human-specific full-length HERV-K.

Type	HERV	Chromosomal position (hg19)	Length (bp)	Comment	Stop codon/Region
			(5'/3'LTR/Internal)		
I	K101	chr 22: 18926187-18935361	968/964/7243	In frame *pol* broken	TGA/*pro*
	K102	chr 1: 155596457-15605636	968/968/7244	In frame *pol*-*env* fusion	TGA/*gag*
	K103	chr 10: 27182399-27183366	968/968/7245	In frame *pol*-*env* fusion/*env* broken	–
	K106	chr 3: 112743124-112752282	960/960/7239	In frame *env* broken	–
	K107	chr 5: 156084717-156093896	968/968/7244	In frame *pol*-*env* fusion	–
	K117	chr 3: 18528336-185289515	968/968/7244	In frame *pol*-*env* fusion/*env* broken	TAG/*env*
	K133	chr 21: 19933659-19941962	966/257/7081	In frame *pol*-*env* fusion/*env* broken	TAG, TGA/*gag*, *pro*, *pol*, *env*
	K134	chr 12: 55727215-55728183	969/968/7243	In frame pol broken	TGA/*pol*
II	K104	chr 5: 30486760-30496205	951/960/7535	–	TGA/*gag*, *pol*, *env*
	K108a	chr 7: 4622057-4631528	968/968/7535	Dual internal sequences, triple LTRs	TAG/*gag*, *env*
	K108b	chr 7: 4630561-4640031	968/968/7535		TAG/*gag*
	K109	chr 6: 78426662-78436083	960/960/7502	–	TAG, TGA/*pol*
	K113	chr 19: 21841536-21841541	968/968/7536	–	–
	K115	chr 8: 7355397-7364859	960/968/7535	–	–
	K118	chr 11: 101565794-101575259	968/968/7530	–	TGA/*gag*, *env*
	K119	chr 12: 58721242-58730698	968/968/7521	–	–
	K121	chr 3: 125609302-125618439	804/804/7530	–	TAG, TGA/*gag*, *pro*, *pol*, *env*
	K132	chr 19: 21841536-21841541	23/995/7869	*Alu* insertion within internal/*pol* broken	–

**Figure 1:**
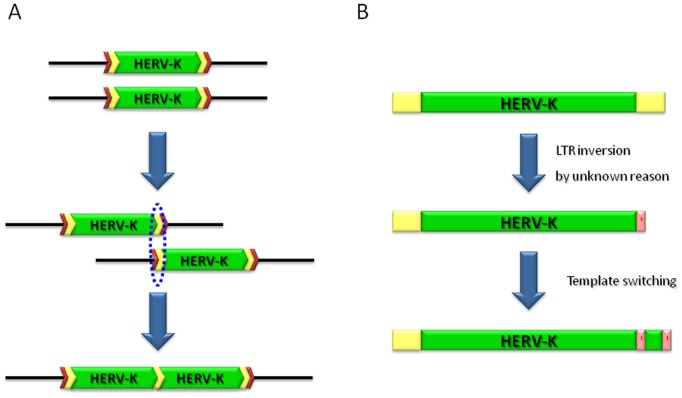
Comparison of human-specific HERV-K108 and HERV-K124 elements. Both of HERV-K108 and HERV-K124 have two HERV-K internal regions (green). However, their sequence architecture is the result of different mechanisms. (A) HERV-K108. After the insertion of the HERV-K element, non-allelic homologous recombination between two different LTRs (yellow chevrons) of the HERV-K element occurred. This resulted in a locus containing two HERV-K internal regions and three LTRs. This locus retains the original TSDs (red chevrons) created upon its initial insertion. (B) HERV-K124. Compared to the HERV-K108, which has two intact internal regions and three intact LTRs, the second internal region of HERV-K124 has largely deleted and its internal and 3′ LTRs inverted and partially deleted. The mechanism(s) responsible for this element’s sequence architecture is not clearly resolved, but we depict here a potential mechanism capable of generating this element. Yellow boxes indicate standard LTRs, pink boxes indicate inverted partial LTRs, and green boxes indicate HERV-K internal regions.

**Figure 2:**
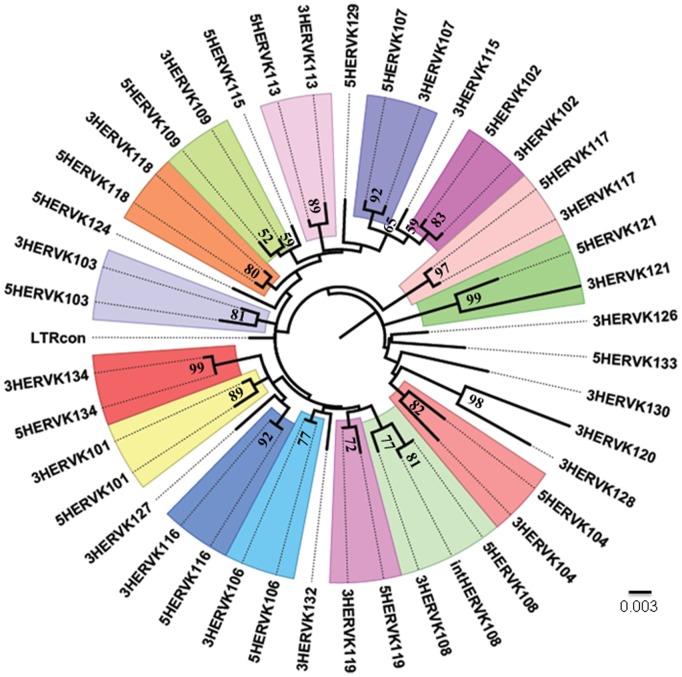
The phylogenetic tree of human-specific HERV-K LTRs. This is a maximum likelihood tree reconstructed using Kimura-2-parameter distance model. Most HERV-K elements contain an LTR at their 5′ and 3′ ends. In cases where the two LTR sequences are similar to one another, they are shown in the same colour. LTRs from the same element but having divergent sequences are not clustered in the same colour. Short LTRs causing ambiguity on this tree were excluded from this analysis. Bootstrap values for nodes (% of 1000 replicates) scoring higher than 50% are reported.

Additionally, we found two interesting human-specific HERV-K loci of non-standard sequence architecture. Each of these consists of two HERV-K internals and three LTRs. One of the two loci, HERV-K108, may have resulted from ectopic homologous recombination between two different LTRs, the mechanism for which was introduced in another study on HERV-K and is depicted in Figure 1A [19]. The three LTRs of HERV-K108 showed a high degree of sequence similarity and were closely related in the phylogenetic tree in Figure 2. The other locus, HERV-K124 also contains three different LTRs. However, it was unclear what mechanism may be responsible for the observed sequence architecture of this locus. If LTR-LTR recombination were to explain this locus, we would expect the three LTRs to have a high degree of sequence similarity to one another, but the 3′ and internal HERV-K124 LTRs are truncated and inverted relative to 5′ HERV-K124 LTR. We therefore speculate that HERV-K124 was generated in two steps: LTR inversion and template switching, as shown in Figure 1B. Although the LTR inversion is a rare event, a possible mechanism responsible for the LTR inversion was suggested in one of previous studies on HERV-K [20].

### Genomic Environment of Human-specific HERV-K Insertions

We aligned the human-specific HERV-K elements based on their LTR sequences except for eight loci because those elements contained LTRs that were too short (23–257 bp) resulting in ambiguity in the alignment. Next, we reconstructed the phylogenetic relationships between these LTRs. It is known that the two LTRs of an HERV element tend to have a high sequence identity to one another. As shown in Figure 2, this expected within-element sequence identity was found in all of our loci except HERV-K115. We suspect that gene conversion may have led to the differences observed between the two LTR sequences of the HERV-K115 [21].

To examine the genomic environment of the human-specific HERV-K insertions, we analyzed the GC content and gene density of genomic regions flanking the elements (Table S1). GC content was calculated for the 20 kb of flanking genomic sequence on each side of each locus. The GC content of these flanking regions averaged 41.6%. This is only slightly higher than the human reference genomic average GC content of 41% [1]. In addition, we analyzed the gene density of the 1 Mb of flanking genomic sequence to each side of the human-specific HERV-K elements and the results are described in Table S1. The gene density of these insertions averaged about 17 genes per Mb, which is substantially higher than the ∼10 genes per Mb average reported for the human genome [1]. It has been previously reported that HERV-K elements are preferentially integrated into GC-rich regions, and thus gene-rich regions [22], and our findings are consistent to with this assertion.

### Polymorphic Distribution of Human-specific HERV-K Insertions

**Figure 3:**
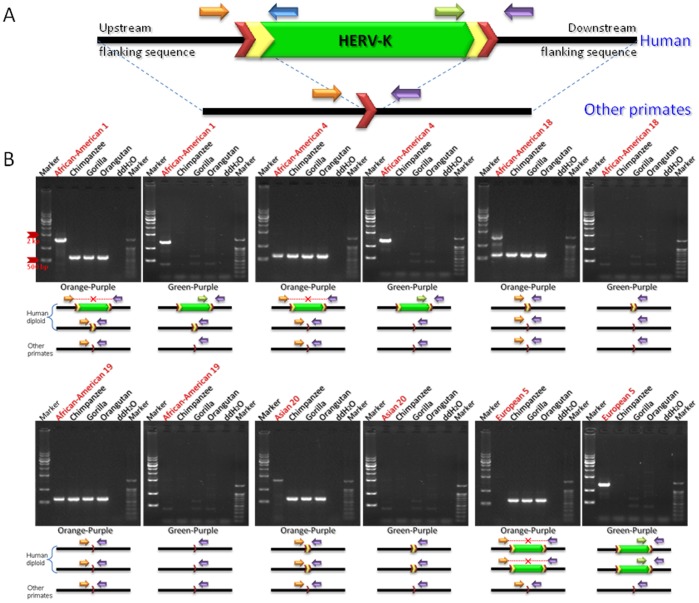
Variable polymorphic patterns of a HERV-K118 in human diploid genomes. Human-specific HERV-K118 insertion locus was amplified by PCR using the genomic DNAs of human population and other primates as template. (A) A typical primate HERV-K element. The ∼7.5 kb structure of the HERV-K internal region is shown in green. Yellow chevrons are LTRs (∼1 kb) and red chevrons are target site duplications (TSDs). (B) Gel chromatographs show PCR products of targeted human-specific HERV-K loci on a panel containing human three non-human primates. High bands indicate the presence of an insertion, while low bands indicate its absence. Orange and purple arrows indicate primers designed in the conserved flanking regions of all species. Green arrows indicate internal primers designed within the human-specific HERV-K. As shown in the gel pictures, human-specific HERV-K insertion loci exhibit a variety polymorphic patterns in human diploid genomes.

The HERV-K family has been shown to be actively mobilizing in the human genome since the divergence of human and chimpanzee, and thus some of these elements are likely to be polymorphic in the human population. To evaluate the polymorphism levels associated with human-specific HERV-K loci, we genotyped 25 loci in 80 humans (20 from Asian, 20 from South American, 20 from European and 20 from African American) whose DNAs were purchased from the Coriell Institute for Medical Research. We were not able to amplify the remaining four loci because they reside either in regions of segmental duplication or in centromeric regions. As shown in Figure 3B, there are three possible states for each sister chromatid at a human-specific HERV-K insertion locus: absence of the HERV-K element, presence of the element and presence of a solitary LTR. Among the human-specific HERV-K elements, three loci, HERV-K 109, 118, and 134, exhibit all the three forms in the human populations tested. The polymorphism test found that the polymorphism level of the human-specific HERV-K elements is about 48% (12/25) which is higher than levels reported for other human-specific retrotransposons [23], [24], [25]. We examined the recombination rate of the genomic regions where the human-specific HERV-K elements reside because a high recombination rate could contribute to the observed increase their polymorphism level. As shown in Table 3, the recombination rates in the genomic regions flanking human-specific HERV-K elements averaged ∼1.2 cM per Mb on both long and short arms. We compared the result with the genome-wide average recombination rates, ∼1 cM and ∼2 cM per Mb on the long and short arms, respectively [1]. Based on the result, we conclude that recombination rate is not a major factor responsible for the higher polymorphism levels observed in human-specific HERV-K elements.

**Table 3:** Characteristic of human-specific HERV-K insertions.

No.	HERV	Genomic location	Features^c^	Rec. rate	size (bp)					Reference
				(cM/Mb; avg)	5' LTR	internal	3' LTR	internal	3' LTR	
1	K116	chr1:75842771-75849143	**Inserted into human-specific L1PA2**	0.7	968	4437	968			[40]
2	K102^a^	chr1:155596457-155605636	**–**	1.3	968	7244	968			[2]
3	K120	chr2:130719538-130722650	**Inserted into SD region**	1.1	23	2129	961			[18]
4	K106^a^	chr3:112743124-112752282	**Polymorphic**	0.4	960	7239	960			[2]
5	K121^a^	chr3:125609302-125618439	**Inserted into SD region**	0.8	804	7530	804			[41]
6	K122	chr3:148281441-148285419	**13 bp L1 sequence in 3' end of ERV, polymorphic**	2.1	23	3920	23			[18]
7	K123	chr3:170955654-170955804	**Non-classical insertion, HERV-K9 subfamily**	1.5	0	143	0			This Study
8	K117^a^	chr3:185280336-185289515	**Inserted into SD region, polymorphic**	2	968	7244	968			[40]
9	K124	chr4:161579938-161582439	**Second HERV-K internal to 206 bp in first HERV-K internal of 3' end**	0.9	968	1171	78^b^	206	78^b^	This Study
10	K104^a^	chr5:30486760-30496205	**–**	1.8	951	7535	960			[2]
11	K107^a^	chr5:156084717-156093896	**Polymorphic**	0.6	968	7244	968			[42]
12	K125	chr6:74042982-74043123	**Non-classical insertion**	0.6	0	142	0			This Study
13	K109^a^	chr6:78426662-78436083	**Polymorphic**	0.7	960	7502	960			[2]
14	K108^a^	chr7:4622057-4640031	**LTR-LTR homologous recombination, polymorphic**	1.6	968	7535	968	7536	968	[2]
15	K126	chr7:104388369-104393269	**13bp L1 sequence in 3' end of ERV**	1.1	0	3921	967^b^			[18]
16	K115^a^	chr8:7355397-7364859	**Inserted into SD region, polymorphic**	0.9	960	7535	968			[30]
17	K127	chr8:140472149-140475259	**–**	2.7	23	2120	968			[18]
18	K128	chr10:101580569-101587739	**–**	0.1	23	6162	968			[16]
19	K118^a^	chr11:101565794-101575259	**Polymorphic**	0.6	968	7530	968			[43]
20	K119^a^	chr12:58721242-58730698	**Polymorphic**	0.3	968	7521	968			[43]
21	K129	chr12:111007843-111009348	**–**	0.6	968	515	23			[44]
22	K130	chr16:34231397-34234142	**Non-classical insertion**	0.1	0	1788	958			This Study
23	K131	chr17:6078917-6079053	**Non-classical insertion**	3.5	0	96	41			This Study
24	K132^a^	chr19:28128498-28137384	**Inserted into satellite DNA region of centromere**	0.4	23	7546	995			[45]
25	K133^a^	chr21:19933659-19941962	**TSD contains partial LTR50, MIRb, and AT_rich**	3	966	7081	257			[46]
26	K101^a^	chr22:18926187-18935361	**Inserted into SD region**	3.3	968	7243	964			[2]
27	K103^a^	chr10:27182399-27183366	**Solitary LTR in hg19, inserted into SD region, polymorphic**	0.9	968	7245	968			[2]
28	K113^a^	chr19:21841536-21841541	**Absence in hg19, inserted into SD region, polymorphic**	0.1	968	7536	968			[30]
29	K134^a^	chr12:55727215-55728183	**Solitary LTR in hg19, polymorphic**	1.1	969	7243	968			[17]

a Full-length human-specific HERV-K locus.

b Sequence is reversed.

c TSD, Target Site Duplication; SD, Segmental Duplication.

Through the polymorphism test, we found that both type I and II full-length human-specific HERV-K elements are polymorphic in the 80 human individuals. This indicates that both types were capable of retrotransposition after the divergence of human and chimpanzee and increases likelihood that members of these groups are currently able to retrotranspose in the human genome.

### Structural Analysis of Human-specific Full-length HERV-K

**Figure 4:**
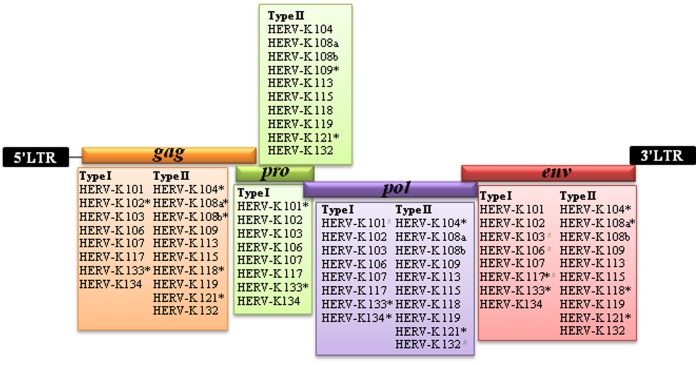
Diagram of a human-specific full-length HERV-K element. The ORFs of *gag*, *pro*, *pol*, and *env* are depicted as colored boxes. HERV-K members that contain versions of *gag*, *pro*, *pol*, and *env* are listed under each HERV genes (* and ^#^ indicate that the HERV-K locus contains stop codon or broken frame, respectively).

The majority of HERVs in the human genome exist in truncated form and are characterized by multiple stop codons, insertions, and deletions [26], [27]. It is suspected that a smaller subset of human-specific HERV-K elements are capable of retrotransposition and thus contain intact open reading frames (ORFs) because their proteins and particles have been detected in the human genome [28]. We therefore examined whether any of the identified human-specific full-length HERV-Ks contain intact ORFs. As shown in Table 2, five human-specific type I HERV-Ks, HERV-K102, 103, 107, 117, and 133, exhibit fused *pol* and *env* genes in the same frame. A search for stop codons in the gene components of the human-specific type I HERV-Ks revealed that HERV-K101, 102, 117, and 134 have stop codons in their *pro*, *gag*, *env*, and *pol* genes, respectively, and HERV-K133 contains stop codons in all of these genes (Figure 4). In sum, a total of three HERV-K elements have retained intact ORFs in the human genome, indicating that they have a potential to produce the viral particles [29].

As mentioned above, the type of HERV-K element is determined according to the presence/absence of a 292 bp ‘deletion’ at the *pol-env* boundary. It has been reported that the ancestral precursor of the type I HERV-K lacked the 292 bp sequence and that this deletion must not have been directly related to the precursor’s ability to retrotranspose in the human genome. This is because the human genome contains at least eight type I full-length HERV-K elements which must be offspring of the precursor [30]. However, we could not rule out other possible origins for Type I insertions. For example, they could result from the recombination between competent Type II viruses and transcripts of preexisting Type I.

Among the human-specific type II HERV-Ks, HERV-K104, 108, 118, and 121 contained stop codons in multiple genes while HERV-K132 had an *Alu* insertion within its *pol* gene. These five HERV-Ks are therefore not functionally and structurally intact in the human genome. However, HERV-K113, 115, and 119 possess intact gene components, which indicates that they have the potential to encode the functional proteins required for their mobilization. HERV-K113 and 115 were previously identified to be full-length and polymorphic (HERV-K presence/absence) in human populations [30]. The result of our polymorphic test on HERV-K119 showed that this element is also polymorphic in the 80 human individuals, but its pattern of polymorphism is different from that of the other elements; the polymorphism at the HERV-K113 and 115 loci takes the form of an absence or presence of the HERV-K element between individuals, but the HERV-K119 locus exists as either a full-length HERV-K or a solitary LTR. We speculate that this architecture is the product of a homologous recombination event between the two LTRs of a full-length HERV-K element. Given this, we suspect that the HERV-K119 element is relatively older than the other two elements (HERV-K113 and 115). These intact full-length HERV-K elements could play a role in human disease. This possibility has been suggested by several reports describing HERV-encoded transcripts and proteins in tumors [31], [32] and tissue from patients with autoimmune diseases [27], [33], [34].

### Human-specific HERV-K Insertion-associated Genetic Variations

**Figure 5:**
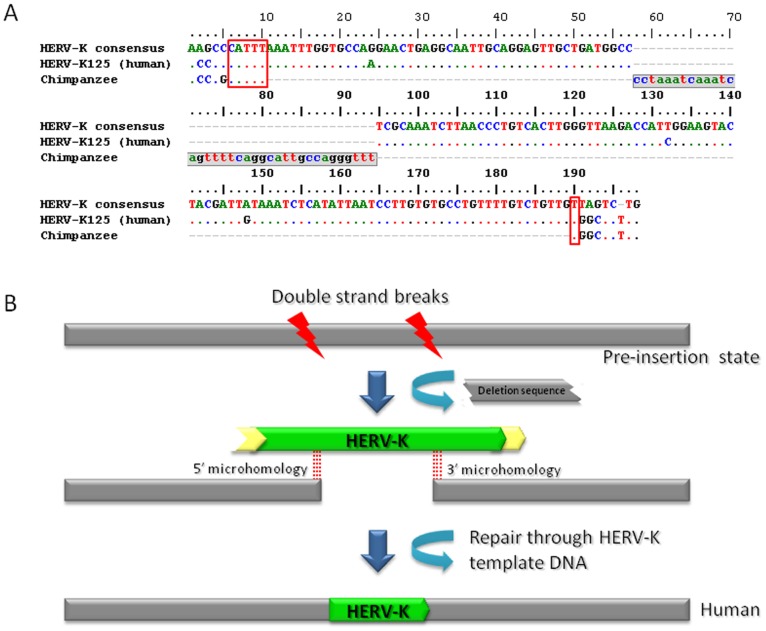
Non-classical insertion of human-specific HERV-K element in the human genome. Four non-classical insertions of human-specific HERV-K were observed in the human genome. The human-specific locus, HERV-K125, is depicted here. (A) An alignment of the non-classical insertion of human-specific HERV-K125 element, and its pre-insertion site to the HERV-K consensus sequence. This alignment reveals a 37 bp deletion of the pre-insertion site in the human genome (gray region in the chimpanzee sequence). Red boxes indicate microhomology at either end of the non-classical insertion, which suggests the involvement of an NHEJ mechanism. (B) A schematic diagram that describes the non-classical insertion of an HERV-K element (green box) and the deleted-region of genomic sequence (broken gray box).

We found four non-classical HERV-K insertion loci in our dataset (Table 1). Figure 5B depicts one possible mechanism responsible for the non-classical insertion. These elements are 5′ and 3′ truncated, meaning that they also do not have classical TSDs. Additionally, they are involved in target site deletions in the human genome. Through a comparison of the human-specific HERV-K flanking sequence and its corresponding chimpanzee pre-insertion sequence, we calculated the deletion size. However, the chimpanzee orthologous sequence of HERV-K130 insertion contained two unsequenced regions. We amplified one of the regions for sequencing (accession number: JQ811903) and the primer sequences are described in Table S2. We estimated the size of the other region using the orangutan reference genome sequence. The deletion sizes of the target sites of the non-classical HERV-Ks range from 6 bp to 10,207 bp. We further examined their genomic environments and found that three of them occurred in intergenic regions and one occurred in an intronic region. It has been reported that non-classical insertions are associated with double-strand break (DSB) repair, a mechanism proposed to aid in stability of fragile sites in the host genome [35]. Also, it has been suggested that DSBs can be repaired through homologous recombination (HR) or non-homologous end joining (NHEJ) to ensure the maintenance of genome integrity in eukaryotic organisms [36]. As for the four non-classical HERV-K insertions, we examined the microhomology between each HERV-K element and its pre-insertion sequence from the chimpanzee genome. Microhomology, if present, could mediate the insertion of the HERV-K between DSB ends via a NHEJ-associated process. We identified 5′ and 3′ microhomologies for three out of the four loci but were not able to detect microhomology for the HERV-K130 locus, as shown in Table S3. We conclude, therefore, in the cases where microhomology exists at both ends of the HERV-K insertion, the likelihood of DSB repair through NHEJ is increased.

In this study, we identified 29 human-specific HERV-K insertions including previously reported three loci (HERV-K103, 113, and 134) that have integrated into the human genome since the divergence of humans and chimpanzees. During this time, HERV-K activity contributed to genomic variation between the two species. Through a polymorphism test, we found that the polymorphic rate of these elements is 48%. This indicates that the activity of the HERV-K family has resulted in genomic variations between and within human populations. It is currently unknown whether there are any retrotranspoitionally competent copies of HERV-K element in the human genome. However, based on the results of this study, we assert that HERV-K element activity is a cause of genomic differences between the human and chimpanzee genomes as well as genomic diversity within the human population.

## Materials and Methods

### Computational Data Mining and Manual Inspection of Human-specific HERV-K Loci

To computationally screen the human genome (hg19; February 2009 freeze) for potential human-specific HERV-K loci, we first extracted all HERV-K loci from the human genome by using UCSC Table Browser utility (http://genome.ucsc.edu/cgi-bin/hgTables?org=Human&db=hg19&hgsid=226995881&hgta_doMainPage=1). For each HERV-K locus, we next extracted 2 kb flanking sequences, up and down stream. This human sequence was then used as a query against other primate genome sequences (panTro3; October. 2010 freeze, ponAbe2; July 2007 freeze, rheMac2; January 2006 freeze), using UCSC’s BLAT utility (http://genome.ucsc.edu/cgi-bin/hgBlat). For each hit in the BLAT search, we retrieved the human, chimpanzee, orangutan and rhesus macaque sequences. Repeat elements existing in these nonhuman sequences were annotated using the RepeatMasker (http://www.repeatmasker.org/cgi-bin/WEBRepeatMasker) tool. Based on these repeat element annotations, we confirmed whether each HERV-K locus was specific to the human genome or not.

### PCR Amplification and DNA Sequence Analysis

To experimentally verify the human-specific HERV-K insertion candidates, we conducted PCR analysis with four different DNA templates: *Homo sapiens* (human; NA10851, Coriell Cell Repository, Camden, NJ), *Pan troglodytes* (common chimpanzee), *Gorilla gorilla* (gorilla), and *Pongo pygmaeus* (Bornean orangutan). Genomic DNA for three apes was kindly provided by Dr. Takenaka (Primate Research Institute, Kyoto University). Oligonucleotide primers for the PCR amplification of human-specific HERV-K insertion candidates were designed, using the Primer3 utility (http://biotools.umassmed.edu/bioapps/primer3_www.cgi) (Table S4). PCR amplification of each locus was performed in 20 µl reaction using 20–30 ng template DNA, 200 nM of each oligonucleotide primer, and 10 µl of EmeraldAmp GT PCR Master Mix (TaKaRa, Ohtsu, Japan). Each sample was subjected to an initial denaturation step of 5 min at 95°C, followed by 35 cycles of PCR at 1 min of denaturation at 95°C, 1 min at the annealing temperature, and 1 to 2 min of extension at 72°C depending on the PCR product size, followed by a final extension step of 10 min at 72°C. The PCR products were loaded on 1–2% agarose gels, stained with ethidium bromide, and visualized using UV fluorescence. For the loci whose expected product size was >2 kb, we used Ex TaqTM polymerase (TaKaRa Japan), 2X EF-Taq Pre mix 2 (SolGent, Korea), and KOD FX (Toyobo, Japan) to carry out PCR following the manufacturer’s instructions.

If needed, we purified PCR products from the agarose gel using the Wizard® SV gel and PCR Clean-up system (Promega) and cloned them into vectors using the pGME®-T Easy Vector system (Promega, http://www.promega.com) according to the manufacturer’s instructions. The sequencing of the PCR product was performed on an ABI 3730xl DNA analyzer (Applied Biobiosystems, www.appliedbiosystems.com) at the oligonucletides synthesis and sequencing facility, MACROGEN (http://dna.macrogen.com/eng). The resulting DNA sequences were analyzed using the BioEdit v.7.0.5.3 sequence alignment software package and have been deposited in Genbank under accession numbers JQ966584-JQ966591 and JQ999963-JQ999964.

### Data Analyses

We downloaded the HERV-K consensus sequence, including LTRs, from the RepeatMasker utility (http://www.repeatmasker.org/cgi-bin/WEBRepeatMasker) and aligned human-specific HERV-K elements with this consensus sequence using the software BioEdit v.7.0.5.3 [37]. To reconstruct the phylogenetic relationships among the human-specific HERV-K elements, we used the software MEGA 5.03 [38]. A maximum likelihood tree based on the observed number of nucleotide differences and a Kimura-2-parameter distance model was built. Each node of the tree was evaluated based on 1000 bootstrap replicates and the percentage of replicates in which each node in the final tree was reconstructed is reported in Figure 2. To examine the GC content of the flanking sequences of the human-specific HERV-K elements, we extracted 20 kb of flanking sequence up and down stream of each element using the Human BLAT search Tool server (http://genome.ucsc.edu/cgi-bin/hgBlat?commend=start). The percentage of GC nucleotides in the flanking sequence was then calculated using the EMBOSS GeeCee server (http://bioweb.pasteur.fr/seqanal/interfaces/geecee.html). For the gene density analysis, we counted the number of genes within a 2 Mb window of flanking sequence centered on each human-specific HERV-K element using the National Center for Biotechnology Information Map Viewer utility (http://www.ncbi.nlm.nih.gov/projects/mapview/map_search.cgi?taxid=9606).

### RetroTector10 Program Application

To determine the genomic structure of human-specific full-length HERV-Ks located on a specific locus, we used the RetroTector10 program (http://www.kvir.uu.se/RetroTector/RetroTectorProject.html) [39]. It contains three basic modules: first, the recognition of LTR candidates; second, the detection of chains of conserved retroviral motifs fulfilling the distance constraints; and third, the attempted reconstruction of the original retroviral protein sequences, combination of the alignment, and properties of the protein ends.

## Supporting Information

Figure S1 **The 29 human-specific HERV-K insertion loci in the human genome.** Blue and green circles indicate the chromosomal locations of full-length and truncated human-specific HERV-K elements, respectively. Among them, 12 loci were polymorphic and 4 loci were non-classical insertions. The karyotype images were created using the idiographica webtool (http://www.ncrna.org/idiographica/).
(PPTX)

Table S1 GC content and gene density in flanking regions of human-specific HERV-K loci.
(XLSX)

Table S2 PCR primers for the sequences deleted by HERV-K130 insertion.
(XLSX)

Table S3 Additional information on human-specific HERV-K insertions.
(XLSX)

Table S4 PCR primers for human-specific HERV-K loci.
(XLSX)
