# Supplementary material for: Human-Specific HERV-K Insertion Causes Genomic Variations in the Human Genome
Source: PLoS One. 2013 Apr 12;8(4):e60605. doi: 10.1371/journal.pone.0060605 (PMC3625200; doi:10.1371/journal.pone.0060605)
Supplement: Figure S1 — The 29 human-specific HERV-K insertion loci in the human genome. Blue and green circles indicate the chromosomal locations of full-length and truncated human-specific HERV-K elements, respectively. Among them, 12 loci were polymorphic and 4 loci were non-classical insertions. The karyotype images were created using the idiographica webtool (http://www.ncrna.org/idiographica/). (PPTX) [file pone.0060605.s001.pptx]

## Slide 1
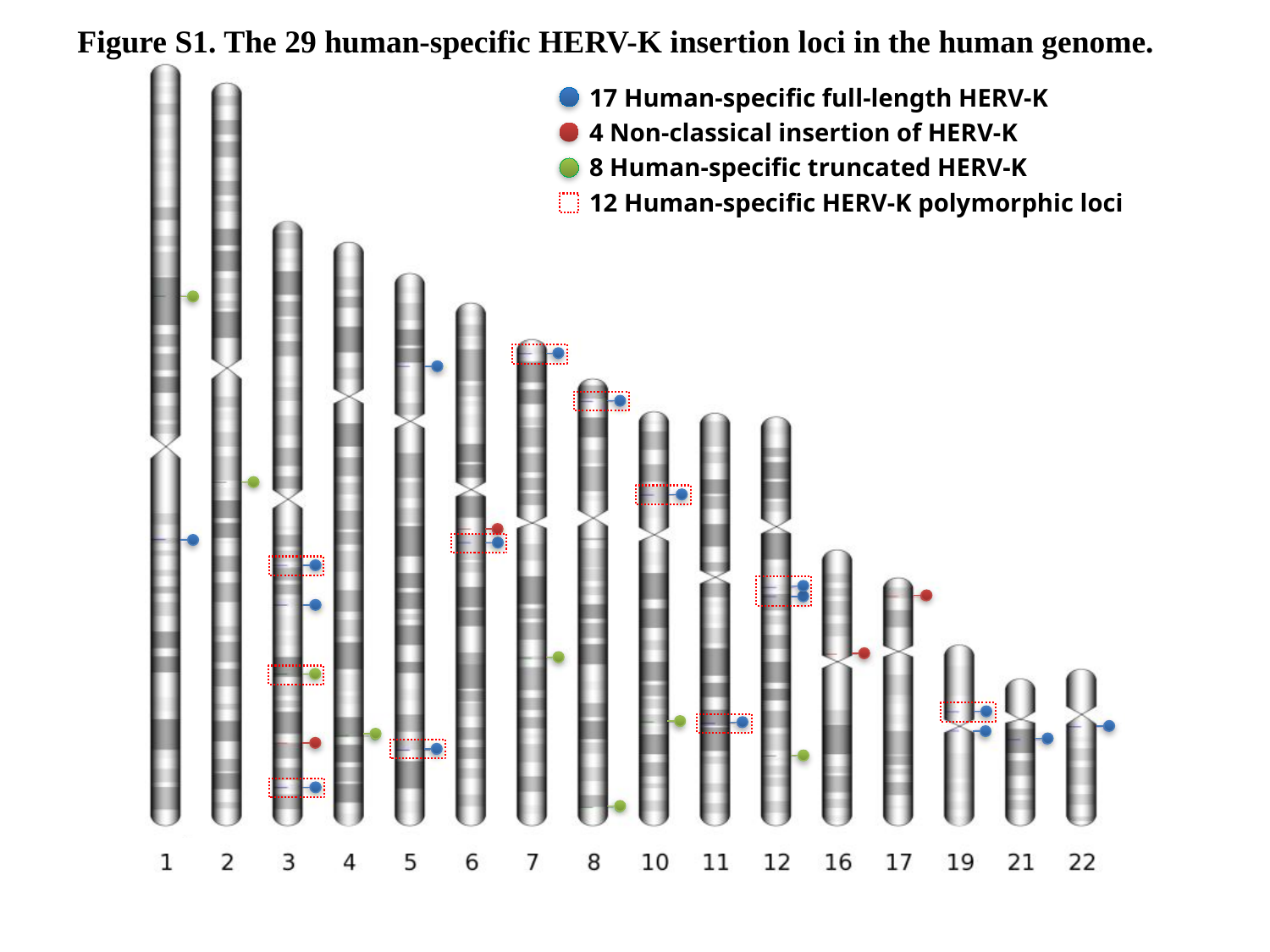

Figure S1. The 29 human-specific HERV-K insertion loci in the human genome.
17 Human-specific full-length HERV-K
4 Non-classical insertion of HERV-K
8 Human-specific truncated HERV-K
12 Human-specific HERV-K polymorphic loci
